# Fatal Exacerbations of Systemic Capillary Leak Syndrome Complicating Coronavirus Disease

**DOI:** 10.3201/eid2710.211155

**Published:** 2021-10

**Authors:** Patricia C. Cheung, A. Robin Eisch, Noble Maleque, Derek M. Polly, Sara C. Auld, Kirk M. Druey

**Affiliations:** Emory University School of Medicine and Rollins School of Public Health, Atlanta, Georgia, USA (P.C. Cheung, N. Maleque, S.C. Auld);; National Institute of Allergy and Infectious Diseases/National Institutes of Health, Bethesda, Maryland, USA (A.R. Eisch, K.M. Druey);; Emory University Hospital Midtown Department of Pharmacy, Atlanta (D.M. Polly)

**Keywords:** capillary leaks, Clarkson disease, coronavirus disease, COVID-19, multiple organ failure, respiratory infections, SARS-COV-2, severe acute respiratory syndrome coronavirus 2, systemic capillary leak syndrome, viruses

## Abstract

We report 2 fatal exacerbations of systemic capillary leak syndrome (SCLS), also known as Clarkson disease, associated with coronavirus disease (COVID-19) in the United States. One patient carried an established diagnosis of SCLS and the other sought treatment for new-onset hypotensive shock, hemoconcentration, and anasarca, classic symptoms indicative of an SCLS flare. Both patients had only mild-to-moderate symptoms of COVID-19. This clinical picture suggests that these patients succumbed to complications of SCLS induced by infection with severe acute respiratory syndrome coronavirus 2. Persons with known or suspected SCLS may be at increased risk for developing a disease flare in the setting of mild-to-moderate COVID-19 infection.

Systemic capillary leak syndrome (SCLS), also known as Clarkson disease, is a rare disease of unknown etiology that most commonly develops in adults 50–70 years of age (*1*). Since SCLS was first characterized in 1960, <500 cases have been described in the medical literature. The current prevalence of SCLS is estimated to be <250 cases worldwide (*1*), although the disease is likely underdiagnosed. 

SCLS is diagnosed clinically on the basis of a characteristic symptomatic triad of hypotension, hemoconcentration (elevated hemoglobin or hematocrit), and serum hypoalbuminemia resulting from fluid extravasation. Patients with SCLS experience transient and reversible episodes of plasma leakage into peripheral tissues, which lead to the acute onset of hypotensive shock and the development of anasarca after intravenous (IV) fluid resuscitation. Severe SCLS flares commonly result in multisystem organ failure and peripheral compartment syndromes (*2*). Between episodes, patients are typically asymptomatic.

Minor infections, typically of the upper respiratory tract, are common triggers for SCLS, although an infection-related prodrome is identified in only 44%–65% of cases (*2*,*3*). Although >80% of SCLS patients have a monoclonal gammopathy of unknown significance (MGUS), the role of this finding in disease pathogenesis remains unclear; the absence of MGUS does not exclude a diagnosis of SCLS (*4*). Interventions for acute SCLS episodes are limited to supportive measures, but monthly prophylaxis with high dose (1–2 g/kg patient weight) IV immunoglobulin (IVIg) prevents attacks among >90% of patients and provides them with a statistically significant survival advantage compared with those patients who were not treated with IVIg (*5*,*6*). Here we report the cases of 2 patients who died of severe SCLS soon after seeking treatment for mild-to-moderate symptoms of COVID-19; neither of them had been receiving IVIg prophylaxis beforehand.

## Methods

Patients were referred to the National Institutes of Health (NIH) for evaluation of suspected SCLS. Where applicable, patients provided written informed consent to participate in a natural history protocol (09-I-0184) approved by the NIH institutional review board. We followed the CARE guidelines for writing medical case reports in the preparation of this manuscript.

### Case Reports

#### Case 1

A 59-year-old woman with a history of hypertension but no known history of SCLS or prior episodes of peripheral edema was admitted to a hospital in January 2021 with a 6-day history of cough, shortness of breath, and lower extremity pain. She was hypotensive, with a blood pressure of 96/70 mm Hg, but her initial physical exam was otherwise unremarkable and included normal results for a pulmonary examination. Laboratory examination revealed hemoconcentration (hemoglobin 17.1 g/dL, hematocrit of 52%) and a lactic acid level of 3.7 mmol/L. Despite resuscitation with 5.5 L Ringer’s lactate and 2.5 L normal saline IV fluids over the first 3 days of admission, serum lactate levels remained elevated at 5.7 mmol/L on day 4 of hospitalization (Table 1). Lower extremity pain worsened, and anasarca without hypoxemia developed. SARS-CoV-2 PCR testing of a nasal swab specimen taken at the time of admission returned positive results; daily treatment with 6 mg dexamethasone and 40 mg enoxaparin was initiated. Remdesivir was not administered because of a developing acute kidney injury. The patient’s lung examination results remained unremarkable, but she became intermittently hypoxemic (SpO_2_ of 88% on 2L nasal cannula), requiring ongoing nasal cannula support. A chest computed tomography revealed bilateral scattered ground glass opacities consistent with mild-to-moderate SARS-CoV-2 infection (Figure). A transthoracic echocardiogram performed on day 3 of hospitalization showed a normal left ventricular ejection fraction (70%–75%), no left ventricular dilation or geometric changes, and no wall motion abnormalities. Other laboratory abnormalities included increased creatinine phosphokinase levels, peaking at 15,094 units/L; elevated lactate at 6.3 mmol/L; and hypoalbuminemia at 2.4 g/dL, which raised concerns for SCLS.

**Table 1:** Laboratory values during hospitalization for case-patient 1 with systemic capillary leak syndrome and coronavirus disease*

Test	Reference range	Admission, 2021 Jan 31	Hospital floor, 2021 Feb 2	Hospital floor/ ICU, 2021 Feb 3	ICU, 2021 Feb 4	ICU, 2021 Feb 5
Albumin	3.5–5.7 g/dL	3.8	3.2	2.4	2.1	2.3
Hemoglobin	11.4–14.4 g/dL	17.1	20.3	17.4	14.5	8.7
Hematocrit	33.3%–41.4%	52.0	61.0	52.6	44.8	26.7
Creatinine	0.6–1.2 mg/dL	0.95	1.2	1.42	1.80	0.85^a^
Sodium	136–145 mmol/L	132	129	126	129	129
Potassium	3.5–5.1 mmol/L	4.3	4.7	5.6	6.1	6.5
Phosphate	2.5–5.0 mg/dL	NA	6.1	5.1	6.9	8.0
Alanine transaminase	7–52 unit/L	32	26	92	299	7,928
Troponin	≤0.04 ng/mL	0.04	<0.03	NA	0.23	0.50
Creatine kinase	30–223 unit/L	NA	1,851	15,094	41,696	>45,000
C-reactive protein	≤10 mg/L	NA	12.8	42.2	41.8	58.1
Leukocytes	4.0–10.0 10^3^/μL	7.0	9.7	19.5	25.3	12.6
Platelets	150–400 10^3^/μL	184	158	145	170	71
Lactate	0.5–2.2 mmol/L	4.6	6.3	5.7	8.2	10.5
D-dimer	≤574 ng/mL	475	NA	1,016	1,271	9,536
International normalized ratio	Not applicable	NA	0.97	NA	1.23	3.29
Activated partial thromboplastin time	26.5–36.5 s	NA	NA	NA	NA	148.9
Complement C3	81–157 mg/dL	NA	NA	NA	74	NA
Complement C4	13–39 mg/dL	NA	NA	NA	22	NA

*ICU, intensive care unit; NA, not available

**Figure:**
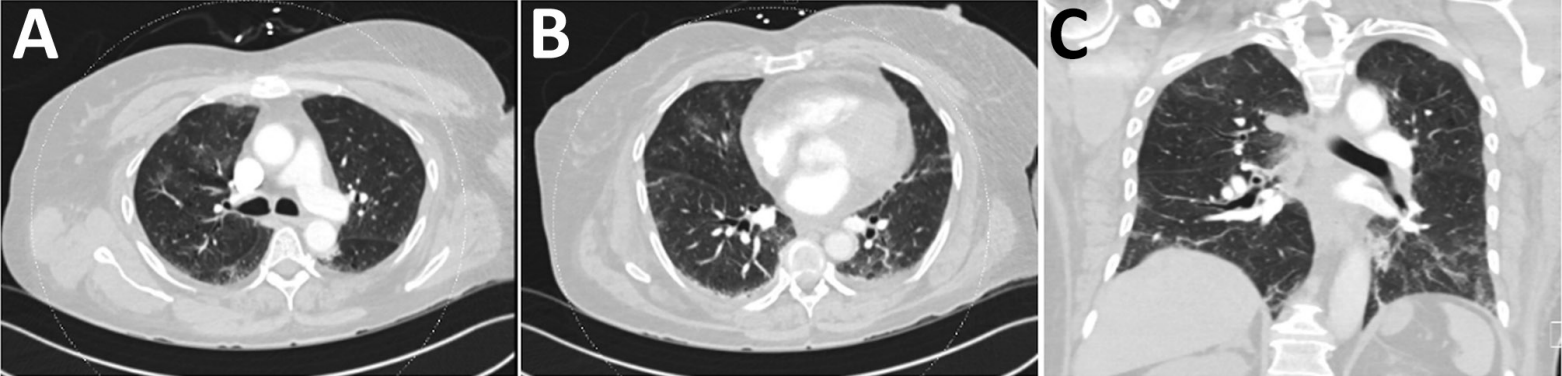
Chest computed tomography from hospital day 3 for a 59-year-old woman (case-patient 1) with new-onset systemic capillary leak syndrome and coronavirus disease showing A) the upper and B) lower lung fields in axial plane and C) the coronal plane. The scans showed bilateral scattered ground glass opacities consistent with mild-to-moderate infection with severe acute respiratory syndrome coronavirus 2.

The patient was transferred to the intensive care unit for further monitoring on day 4 of hospitalization. Pain increased in her extremities and tense anasarca developed; however, because compartment pressures were 18 mm Hg in her right arm and 15 mm Hg in her left arm, she did not meet criteria for a diagnosis of compartment syndrome. Apart from edema and tachypnea while on 2 L nasal cannula, her cardiopulmonary examination was otherwise normal. On hospitalization day 5, she suffered a cardiac arrest; spontaneous circulation returned after 2 rounds of cardiopulmonary resuscitation. She was intubated at the time of cardiac arrest and empirically started on vancomycin and cefepime. Because of concern for microvascular thrombi in the setting of SARS-CoV-2 infection, an argatroban infusion was started. Although the patient had remained afebrile and hemodynamically stable up to this point, shock rapidly developed, and she required vasopressor support with norepinephrine, vasopressin, epinephrine, and stress-doses of hydrocortisone. Continuous renal replacement therapy was initiated for oliguric renal failure.

The next day, edema in the extremities intensified, and creatinine phosphokinase levels increased further to >45,000 units/L, but compartment pressures were 17 mm Hg in her right arm and 16 mm Hg in her left arm, suggestive of rhabdomyolysis without compartment syndrome. No MGUS was detected. Serum SARS-CoV-2 IgG was not detected, prompting treatment with convalescent plasma. Given the patient’s grave condition and lack of proven interventions for acute SCLS, empiric treatments were also administered, including IVIg; methylene blue, an agent that suppresses downstream effects of nitric oxide (*7*); and icatibant, a bradykinin receptor antagonist used to treat vascular leakage associated with hantavirus infection (*8*). Bradycardia developed and ultimately required transvenous pacing. Within hours, her hemoglobin decreased to 6.8 g/dL although there was no obvious source of hemorrhage. Because she was anticoagulated with argatroban and had a prolonged activated partial thromboplastin time in the setting of acute liver failure, the patient was transfused with packed erythrocytes. She suffered cardiac arrest, and ventricular fibrillation deteriorated into pulseless electrical activity. Transthoracic echocardiography performed during ACLS revealed no pericardial effusion. The patient received additional units of erythrocytes, IV fluids, fresh frozen plasma, and prothrombin complex concentrate during the cardiac arrest. However, spontaneous circulation was not restored, and the patient died on day 6 of her hospitalization.

#### Case 2

A 36-year-old man with no notable medical history first sought treatment in 2015 for transient hypotension and severe bilateral lower-extremity edema; laboratory testing showed hemoconcentration (hemoglobin 20.2 g/dL, hemocrit 59.4%) and a serum hypoalbuminemia level of 2.2 g/dL after several days of fevers and upper respiratory symptoms. SCLS was diagnosed on the basis of characteristic clinical presentation and an IgG lambda MGUS. His course was complicated by acute kidney injury and compartment syndromes in both legs, which required bilateral fasciotomies. He also had deep vein thromboses in the right internal jugular and left cephalic veins. He was treated transiently with anticoagulants, but a full evaluation for hypercoagulability was negative. Muscle biopsies taken at the time of fasciotomies provided no evidence of inflammatory myositis. All symptoms resolved before he was discharged from the hospital, although a bilateral sensorimotor neuropathy developed, presumably as a residual effect of compartment syndrome.

The patient was treated with IVIg (2 g/kg) within 24 hours of the 2015 hospitalization and monthly thereafter with no recurrence of his SCLS-related symptoms. In November 2016, IVIg prophylaxis was discontinued at the patient’s request. In 2017 and 2018, the patient experienced 2 episodes of bilateral lower extremity swelling, in both cases after several days of upper respiratory symptoms. Blood pressure and laboratory tests were normal at the time of these episodes, and swelling resolved without further treatment.

In February 2020, the patient sought treatment for a several-day history of fevers (40°C) and productive cough. He was noted to be hypoxemic (SpO_2_ of 87% on room air). *Mycoplasma* pneumonia was diagnosed on the basis of chest radiographic evidence of lung infiltrates and positive *Mycoplasma* serologic testing. He was hospitalized and treated with IV antimicrobial drugs and fluids (10.5 L total). Although the fevers and respiratory symptoms resolved, bilateral leg swelling and serum hypoalbuminemia developed, prompting treatment with 1 dose of IVIg (2 g/kg) for empirically presumed SCLS-related edema in the absence of any other proven treatment options. He ultimately recovered and experienced no residual symptoms.

In January 2021, the patient was transported to the emergency department because of disorientation after several days of upper respiratory symptoms, fever (40.6°C), and back pain. Upon arrival, he was hypotensive, with a blood pressure of 94/20 mm Hg, and tachycardic at 140 beats/min. Upon initial examination, he showed no signs of apparent dyspnea (respiratory rate of 19 breaths/min) or respiratory distress (SpO_2_ of 99% on room air). A PCR nasal swab test for SARS-CoV-2 performed on the day before he sought treatment was reported by the family to be positive. Laboratory tests taken at the time of hospital admission revealed severe hemoconcentration (hemoglobin >25 g/dL, 75% hemocrit), leukocytosis (leukocyte count 43.3K/μL blood), and lactic acidosis (lactate level 9.2 mmol/L). Other laboratory values included brain natriuretic peptide at 12 pg/mL, troponin at <0.02 ng/mL, and a fingerstick glucose level of 197 mg/dL. There was no ST elevation suggesting myocardial infarction or dysrhythmia on his electrocardiogram. 

In the emergency department, his condition deteriorated rapidly. His blood pressure became unobtainable manually, and he exhibited an Sp0_2_ of 73% on a 15-L nonrebreather mask. A bedside echocardiogram revealed a flat inferior vena cava, intact bilateral ventricular function, and pericardial effusion with no evidence of right heart strain or tamponade. He was intubated emergently but ultimately experienced cardiac arrest with pulseless electrical activity. Despite aggressive cardiopulmonary resuscitation and pharmacological interventions including boluses of epinephrine, bicarbonate, calcium, and magnesium, refractory ventricular tachycardia developed, followed by Torsade de Pointes, and he died shortly thereafter.

## Discussion

We report 2 cases of SCLS associated with mild-to-moderate COVID-19 infection. Case-patient 1 exhibited the clinical diagnostic triad for SCLS: hypotension, hemoconcentration, and hypoalbuminemia. Case-patient 2 carried a prior diagnosis of SCLS. These findings suggest that patients with SCLS may be at high risk for exacerbations or even death if they contract COVID-19.

Information from our studies complements the findings of several recent case reports of severe SCLS attacks associated with mild-to-moderate SARS-CoV-2 infection (*9*–*12*). In each of the cases in those studies, patients exhibited all the hallmarks of severe SCLS exacerbations after experiencing mild-to-moderate symptoms of COVID-19; 2 of 3 patients died from SCLS-related complications.

Because COVID-19 had not previously been associated with secondary capillary leak syndrome and there were no other obvious triggers for the episode in case-patient 1 in our study, it is highly likely that she carried latent SCLS even though MGUS was not detected at the time of the episode. Although MGUS is an important clue, it is not detected in all patients with SCLS. In the most recent comprehensive reviews of the literature, which included 290 cases reported during 1960–2016, MGUS was detected in only ≈75% of case-patients (*3*). Although MGUS was detected in 34 (91%) of 37 patients in a 2017 study (*2*), the authors emphasized that “the three monoclonal gammopathy-negative patients had typical severe SCLS flares.” Several studies failed to establish any functional role for these monoclonal paraproteins in disease pathogenesis (*13*–*15*). Finally, immunofixation may be negative during acute SCLS flares because of IgG extravasation and transient hypogammaglobulinemia (*15*,*16*).

Several unique clinical and laboratory features of SCLS can be used to differentiate disease flares from the sequelae of severe COVID-19 (Table 2). Most notably, edema of the trunk and extremities is a prominent feature of acute SCLS and can lead to the development of compartment syndromes. Edema in COVID-19 infection is typically peripheral and frequently confined to the fingers and toes in association with chilblains, painful erythematous lesions (*17*). Pulmonary edema is a feature of SCLS rarely noted at initial observation but is a frequent characteristic of acute COVID-19–associated acute respiratory distress syndrome (*18*). Furthermore, both patients exhibited severe hemoconcentration despite aggressive fluid resuscitation. Patients who are critically ill with COVID-19 typically exhibit anemia, which is a predictor of a poor clinical outcome (*19*). Finally, although mildly decreased serum albumin levels of ≈3 g/dL have been reported in patients with severe COVID-19 (*20*), hypoalbuminemia is typically much more severe in SCLS flares, with an albumin level usually <2 g/dL.

**Table 2:** Clinical characteristics of acute SCLS compared with severe COVID-19*

Parameter	SCLS	COVID-19
Blood pressure	Low/undetectable, vasopressor-resistant	Normal/high
Hgb/Hct	Very elevated	Normal/low
Serum albumin	Very low	Normal/low
Pulmonary findings	Absent	Lung infiltrates, tachypnea, hypoxemia
Edema	Anasarca, compartment syndrome	Pulmonary edema; no peripheral edema
Creatine kinase	Very elevated	Normal/elevated
Creatinine	Elevated, acute kidney injury common	Normal

*COVID-19, coronavirus disease; Hgb/Hct, hemoglobin/hematocrit ratio; SCLS, systemic capillary leak syndrome.

Of note, SCLS had not previously been diagnosed in case-patient 1, and neither patient was receiving IVIg prophylaxis at the time of COVID-19 infection. Fortunately, we observed no COVID-19–associated SCLS flares in any of the >70 patients in the study cohort who were receiving IVIg prophylaxis (range 0.75–2.00 g/kg/mo). However, a previously published report (*11*) documented the case of a patient whose disease had been well-controlled by IVIg prophylaxis (0.5 g/kg/mo) but who died of refractory SCLS soon after COVID-19 infection. Nonetheless, in agreement with the authors of that study, we strongly recommend that SCLS patients receive IVIg prophylaxis indefinitely, at the highest recommended dose (2 g/kg/month), until the COVID-19 pandemic is under better control. Because no treatments for acute SCLS flares, including IVIg, have been proven effective, interventions are limited to supportive measures such as IV fluids and albumin, vasopressors, renal replacement therapy, and intubation. However, as noted in recent surveys of critically ill SCLS patients (*2*), fluid administration must be limited to avoid development of compartment syndromes and limb ischemia. Although case-patient 1 received several empiric treatments, including IVIg, icatibant, and methylene blue, the treatments appeared to have had no effect on her intermediate clinical outcomes.

Although the genetic basis of SCLS is not well understood (*21*,*22*), our work has provided evidence that patients with SCLS experience intrinsically exaggerated endothelial barrier dysfunction in response to otherwise mundane proinflammatory mediators (*23*,*24*). Severe COVID-19 and acute SCLS are both characterized by transient increases in the levels of proinflammatory cytokines in circulation; some of these cytokines, including C-X-C motif chemokine ligand 10, C-C motif chemokine ligand 2 and 3, interleukin -6, and tumor necrosis factor–α (Table 3), directly provoke endothelial barrier disruption (*25*–*27*). These results suggest that the cytokine storm associated with mild-to-moderate COVID-19 may lead to an SCLS flare. Alternatively, as suggested elsewhere (*9*), it is also possible that SARS-CoV-2 may be directly toxic to endothelial cells. This hypothesis suggests that viral factors synergize with host-intrinsic mechanisms to provoke severe SCLS flares. Endothelial cell infection, diffuse inflammatory endothelitis, and microvascular thrombosis are common in COVID-19, although these responses are typically associated with prominent lung involvement (*28*). Because neither of these patients exhibited prominent pulmonary abnormalities, these mechanisms may not be substantial components of COVID-19–associated SCLS. 

**Table 3:** Typical serum cytokine profiles for acute SCLS compared with severe COVID-19*

Cytokine	SCLS	COVID-19
IL-2	Normal	Elevated
IL-4	Normal	Elevated
IL-6	Elevated	Elevated
IL-7	Normal	Elevated
IL-10	Normal	Elevated
CXCL10	Elevated	Elevated
CCL2	Elevated	Elevated
TNF-α	Variable	Elevated
IFN-γ	Elevated	Elevated

*COVID-19, coronavirus disease; CCL, C-C motif chemokine ligand; CXCL, C-X-C motif chemokine ligand; IFN, interferon; IL, interleukin; SCLS, systemic capillary leak syndrome; TNF, tumor necrosis factor.

Further characterization of the immune and inflammatory responses to SARS-CoV-2 will be needed to elucidate its effects on SCLS pathophysiology at the molecular level. However, clinicians should be aware that patients carrying a diagnosis of SCLS or another relapsing-remitting and inflammation-related disease (e.g., autoimmune or autoinflammatory rheumatological diseases) may be at increased risk for severe disease and require increased vigilance for this rare but potentially fatal potential complication of COVID-19 as the pandemic continues.
